# Carbonyl Oxide Stabilization from Trans Alkene and Terpene Ozonolysis

**DOI:** 10.1021/acs.jpca.3c03650

**Published:** 2023-10-04

**Authors:** Jani Hakala, Neil M. Donahue

**Affiliations:** † Center for Atmospheric Particle Studies, Carnegie Mellon University, Pittsburgh, Pennsylvania 15213, United States; ‡ Institute for Atmospheric and Earth System Research, Department of Physics, University of Helsinki, P.O. Box 64, Helsinki, 00014, Finland

## Abstract

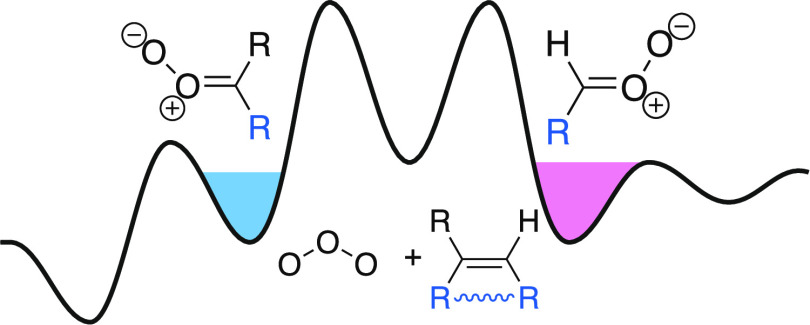

The pressure dependence of carbonyl oxide (Criegee intermediate) stabilization can be measured via H_2_SO_4_ detection using chemical ionization mass spectrometry. By selectively scavenging OH radicals in a flow reactor containing an alkene, O_3_, and SO_2_, we measure an H_2_SO_4_ ratio related to the Criegee intermediate stabilization, and by performing experiments at multiple pressures, we constrain the pressure dependence of the stabilization. Here, we present results from a set of monoterpenes as well as isoprene, along with previously published results from tetramethylethylene and a sequence of symmetrical trans alkenes. We are able to reproduce the observations with a physically sensible set of parameters related to standard pressure falloff functions, providing both a consistent picture of the reaction dynamics and a method to describe the pressure stabilization following ozonolysis of all alkenes under a wide range of atmospheric conditions.

## Introduction

Ozonolysis of alkenes is the canonical 1,3-dipolar cycloaddition reaction.^1^ For many years, the structure of the so-called Criegee intermediate was uncertain, but it is now well established that the intermediates are carbonyl oxides, with a primarily zwitterionic character.^2^ Gas-phase ozonolysis has also been recognized as an important source of atmospheric oxidants, including hydroxyl radicals, OH.^3−5^ Under some conditions, this can be an important atmospheric OH source.^6^ However, a key aspect of gas-phase ozonolysis is the extremely large exothermicity of more than 350 kJ mol^–1^. This leads to nascent reactant products with a high degree of rovibronic excitation, known as chemical activation,^7,8^ which can cause a high degree of fragmentation among reaction products, such as the so-called “hot acid” pathway.^4^ The high excitation also means that collisions with the bath gas are essential to the stabilization of any reaction products.

Two major conformers of Criegee intermediates have notably different chemistries. When the terminal oxygen of the C–O–O moiety faces an α carbon, they are known as syn-intermediates; when the terminal oxygen faces an α hydrogen, they are known as anti-intermediates.^2,5,9^ Because of the conjugation associated with the zwitterionic character of the planar C–O–O moiety, the barrier for interconversion between these conformers is high and effectively insurmountable under atmospheric conditions.^10^ Under most circumstances, the syn-intermediates can abstract an H atom from the α-carbon in the R group to form a closed-shell (but weakly bound) vinyl hydroperoxide.^9,11^ This then decomposes to produce OH radicals with nearly 100% yield, although for more complex vinyl hydroperoxides, OH roaming is an interesting but relatively minor (<10%) pathway.^12^ The low-energy pathway for anti-intermediates is ring closure in the C–O–O moiety to form a dioxirane, which subsequently reopens to form a bisoxy O–C–O biradical. This in turn decays into a hot acid with multiple low-energy fragmentation pathways, forming OH radicals with a roughly 15% yield.^13^ These conformers also have different thermal decay rates and reactivity with key species including H_2_O and SO_2_.^5,14,15^

The Criegee intermediates may also participate in bimolecular reactions if they form so-called stabilized Criegee intermediates (sCIs), either via collisional stabilization or because they are “born cold” below the critical dissociation energy. Collisional stabilization depends on pressure, as in all unimolecular reactions. Stabilization depends critically on both the size (number of internal modes) of the Criegee intermediate and the internal energy (chemical activation). However, even once stabilized, at room temperature, sCIs also have thermal lifetimes of a few seconds or less.^16−18^ The gas-phase chemistry of these sCI depends strongly on their conformation.^15,19,20^ For example, many anti–sCIs react relatively rapidly with H_2_O^5,21,22^ and relatively slowly with other species including SO_2_; in contrast, the syn sCIs react rapidly with SO_2_ and slowly with H_2_O.^5,23^

Criegee intermediate stabilization also plays a key role in atmospheric new particle formation and growth.^24−26^ On the one hand, the sCI can be a highly selective source of gas-phase H_2_SO_4_, which is an important driver of new particle formation.^27^ On the other hand, peroxy radicals derived from CI decomposition products appear to be especially effective initiators of RO_2_ autoxidation,^24,28^ which is in turn the source of the so-called “highly oxygenated organic molecules” (HOM), that are key drivers of organic new particle formation and growth.^24,29,30^ In this case, bimolecular reactions of sCIs may inhibit organic nucleation and growth by intercepting the organics before HOM formation.

In the past decade, techniques have emerged to synthesize Criegee intermediates without the excitation produced from ozonolysis.^31,32^ This has led to tremendous advances in our understanding of Criegee intermediates, as it permits both thermal kinetics and state-resolved experiments.^33^ However, as far as we know, the major source of Criegee intermediates in the atmosphere, including sCI, is ozonolysis. Consequently, it remains important to understand the fundamentally interesting collisional stabilization following ozonolysis reactions.^34^

Here, we extend measurements we have reported previously on symmetrical model systems (tetramethylethylene and trans alkenes)^35,36^ to atmospherically important biogenic terpenoids including monoterpenes and isoprene. Furthermore, by interpreting the pressure dependence of the full suite of experimental results, we present a general description of Criegee intermediate stabilization that promises to extend to the gas-phase ozonolysis of all alkenes.

## Essential Concepts

### Gas-Phase Ozonolysis

Ozonolysis initially forms a primary ozonide (POZ, a 1,2,3–trioxolane), which rapidly decomposes in a cycloreversion to form vibrationally excited carbonyl oxides (Criegee intermediates) along with carbonyl coproducts. Here, we focus on gas-phase systems, where POZ decomposition is rapid, and also assume that concerted scission of C–C and O–O bonds gives bimolecular (or for endocyclic alkenes, unimolecular) products with unit yields.^8,37^ Some computational calculations have suggested that stepwise POZ decomposition, initially via O–O scission, may also contribute to systems such as ethene + ozone,^38^ but to the best of our knowledge, there is little direct experimental evidence for this pathway.

The experiments described here rely on oxidizing SO_2_ to H_2_SO_4_ with both OH and sCI, with and without an added OH scavenger to remove any OH before it otherwise reacts with SO_2_. Our experimental signal is the subsequent measurement of the H_2_SO_4_ product with NO_3_^–^ chemical ionization mass spectrometry and specifically the ratio with and without the additional OH scavenger. This does not require accurate calibration of H_2_SO_4_ measurements because it relies on the ratio of the signal without and with the added OH scavenger.^35^ However, we do rely on independent knowledge of the syn:anti ratios as well as the OH yields from each when they are vibrationally excited.

We assume that an alkene (Ene) forms excited Criegee intermediates (eCIs) with some stoichiometric yield, α^CI^, along with other oxidized organic products (OxOrg). The Criegee intermediates can decay to produce OH radicals, again with some stoichiometric yield α^OH^, but they may also be collisionally stabilized to stabilized criegee intermediates, sCIs, with a stoichiometric yield α^sCI^ that will be a strong function of pressure (bath gas collision frequency). The nominal reaction sequence is
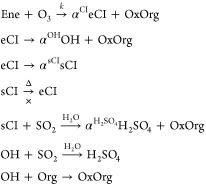
The production rate of sCI is
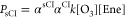
With sufficient SO_2_ to prevent any sCI decomposition (reactivation to eCI, which is excluded with the “×” above), the production rate of OH is
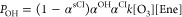
With the OH scavenger present, the production rate of H_2_SO_4_ is
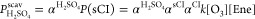
Without the OH scavenger, the production rate of H_2_SO_4_ is
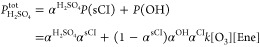
Provided that there is sufficient SO_2_ for H_2_SO_4_ formation to integrate both of these production terms completely, the H_2_SO_4_ signal ratio is equal to the ratio of production rates with and without the OH scavenger
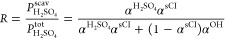
1If H_2_SO_4_ loss is first order and constant (i.e., wall loss), the measured signal *S*_H_2_SO_4__ ∝ *P*_H_2_SO_4__ and this ratio applies to any measured H_2_SO_4_ signal as well.

The reaction of sCI and SO_2_ proceeds through an ozonide intermediate that could itself be stabilized rather than forming SO_3_.^39,40^ However, experiments explicitly measuring production of highly oxygenated organic molecules from α-pinene + O_3_ in the presence of SO_2_, including determination of sCI yields, did not observe clusters containing HOMs and sulfur.^26^ Therefore, here, we assume α^H_2_SO_4_^ = 1.

### Pressure Dependence of Stabilization

The Criegee intermediate stabilization will increase with pressure and we assume that the pressure dependence can be described by a general pressure-dependent falloff curve given by a modified Lindemann–Hinshelwood expression based on numerical solutions to RRK theory^41^
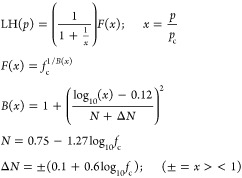
2The parameters are the center of the falloff curve, *p*_c_, which is the intersection point between the linear low-pressure limit and a broadening term, *f*_c_. The expression scales with a nondimensional pressure, *x* = *p*/*p*_c_, and for a pure Lindemann–Hinshelwood reaction, it is 0.5 at *x* = 1. In general, this is true if the lifetime of an excited species is uniform with energy above some stabilization threshold. When the lifetime decreases substantially with energy, the resulting falloff curve is broadened, and the overall Kassel integrals, which are themselves numerical solutions to RRK theory, are reasonably represented by the broadening factor shown, with a broadening factor 0 < *f*_c_ ≤ 1. For *x* = 1, the factor is *f*_c_, so this gives the amount the stabilization is lowered by at the characteristic center of the falloff curve; the broadening term relaxes symmetrically (in the log *x* space) back toward 1.0 away from this central point. The full form of these approximate analytical fits to the Kassel integrals is needed when *f*_c_ ≪ 0.5, such as for the OH + NO_2_ reaction.^42^ The common gas-phase kinetic compilations use simplified expressions, either with *B*(*x*) = 1 + (log_10_(*x*))^2^^43^ or with *f*_c_ = 0.6 as well.^44^ Because the broadening is caused by variation in the excited-state lifetime of the species being stabilized, for these systems with high chemical activation, we expect substantial broadening, with *f*_c_ < 0.5, and so employ the full functional approximations.

The Tröe function was developed for association reactions and at least semistrong collisions (with a single collision providing some stabilization).^41^ For ozonolysis, we have high chemical activation and fragmentation in the products, and so we model pressure stabilization of each Criegee intermediate with the modified version of that function
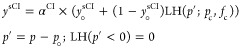
3We allow the sCI yields to have either a zero-pressure *y*-intercept, *y*_°_^sCI^, or an *x*-intercept “induction pressure”, *p*_°_. The *y*-intercept occurs when a fraction of the nascent Criegee intermediates are “born cold” with internal energy below the critical decomposition energy.^16^ The *x*-intercept induction pressure occurs when the entire nascent energy distribution is far above that critical energy, and it takes multiple collisions before any appreciable stabilization occurs. Previous master equation simulations of substituted cyclohexenes show that until the collision frequency (pressure) reaches a value within a few orders of magnitude of the unimolecular decomposition rate coefficient, the stabilization yield is negligible.^37^ At very low pressure, rather than being linear with pressure, stabilization yields are between second and third order in pressure, and here, we approximate this behavior with the *x*-intercept when needed.

The yield of stabilized Criegee intermediates, sCIs, of each type (syn, anti, etc.) is given by an individual falloff expression. This leaves the residual as the yield of the excited Criegee intermediate, eCI, and for a reaction with multiple types of Criegee intermediate, the fraction of each type comprising the eCI population is critically important. Specifically, it means that the overall OH yield from excited intermediates depends on stabilization and thus pressure.
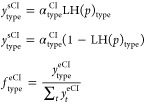
In this scheme with ample SO_2_ to scavenge sCI, the yield of OH is only from excited Criegee intermediates.
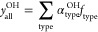
4This complicates the data interpretation.

### Examples

Key aspects can be illustrated with examples. For all these examples, we shall consider ozonolysis, producing excited Criegee intermediates with no intercepts and overall stabilization parameters *p*_c_ = 80 Torr and *f*_c_ = 0.275, meaning a very broad falloff with the center of the falloff curve at 80 Torr. We shall consider several cases, all with identical actual stabilization yields given by this function but with quite different H_2_SO_4_ signal ratios.

#### All Syn

**Figure 1:**
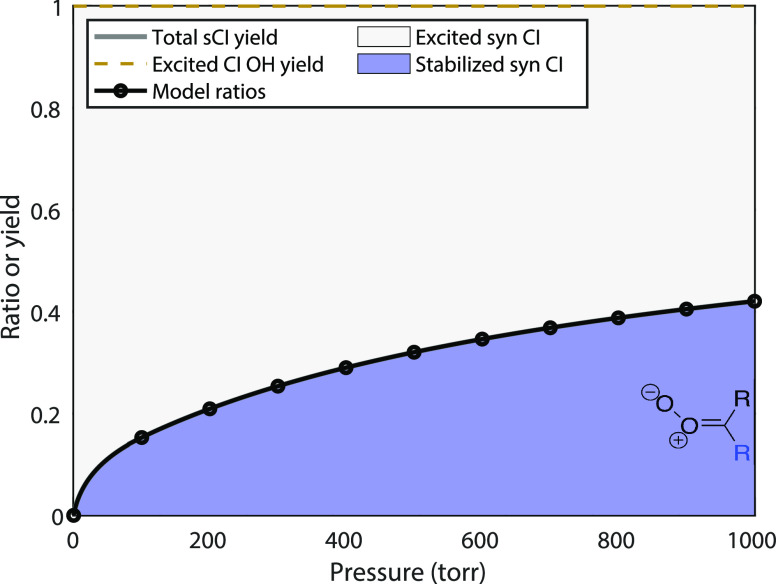
Stabilized Criegee intermediate yields, sCI, and H_2_SO_4_ signal ratios, R = *S*_scav_/*S*_tot_, for a fully symmetrical, all syn-intermediate with *y*^OH^ = 1.

The simplest case is when all Criegee intermediates are syntactic and produce OH with *y*^OH^ = 1. An example is fully symmetrical 2,3–dimethyl–2-butene (tetramethylethylene (TME)). As shown in Figure 1, the measured signal ratio and the actual sCI yields are identical, and with these parameters, the yield rises to *y*^sCI^ ≃ 0.4 near 1000 Torr. The extensive broadening associated with *f*_c_ = 0.275 causes a steep rise for *p* < 100 Torr followed by a steady rise in yield toward higher pressure.

#### All Anti

**Figure 2:**
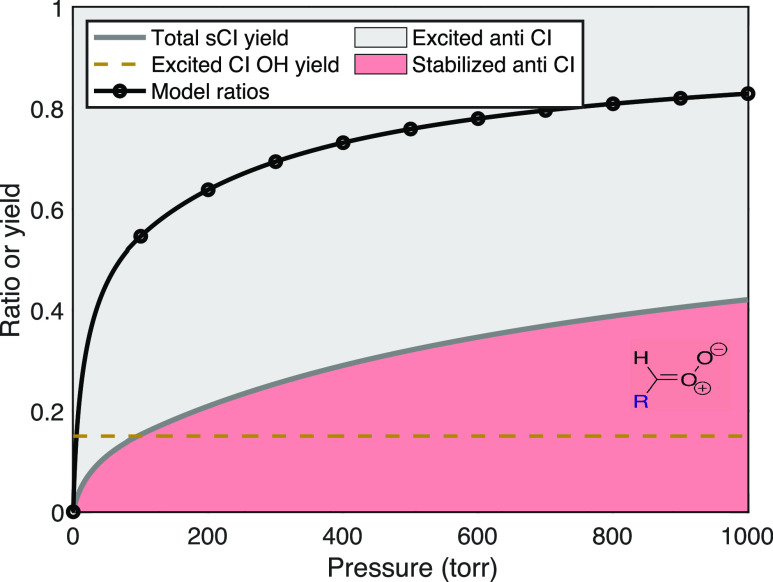
Stabilized Criegee intermediate yields, sCI, and H_2_SO_4_ signal ratios, *S*_scav_/*S*_tot_, for a fully symmetrical, all anti-intermediate with *y*^OH^ = 0.15.

The second simplest case is when all Criegee intermediates are identical and anti and produce OH with a much lower (constant) yield, *y*^OH^ = 0.15. Almost the only real-world example is ethene, producing formaldehyde oxide CH_2_OO. As shown in Figure 2, the expected H_2_SO_4_ signal ratio and the actual sCI yields are now very different. For the purposes of illustration, the pressure-dependent yield is for this example, the same as in the first example (shown in each case with the colored region and gray curve). The signal contrast is much lower when an OH scavenger is added because *y*^OH^ is so low. Low signal contrast is a high ratio (near 1.0), and so the observed ratios in Figure 2 are much greater than the actual yields.

#### Mixture of *syn*- and *anti*-Conformers

**Figure 3:**
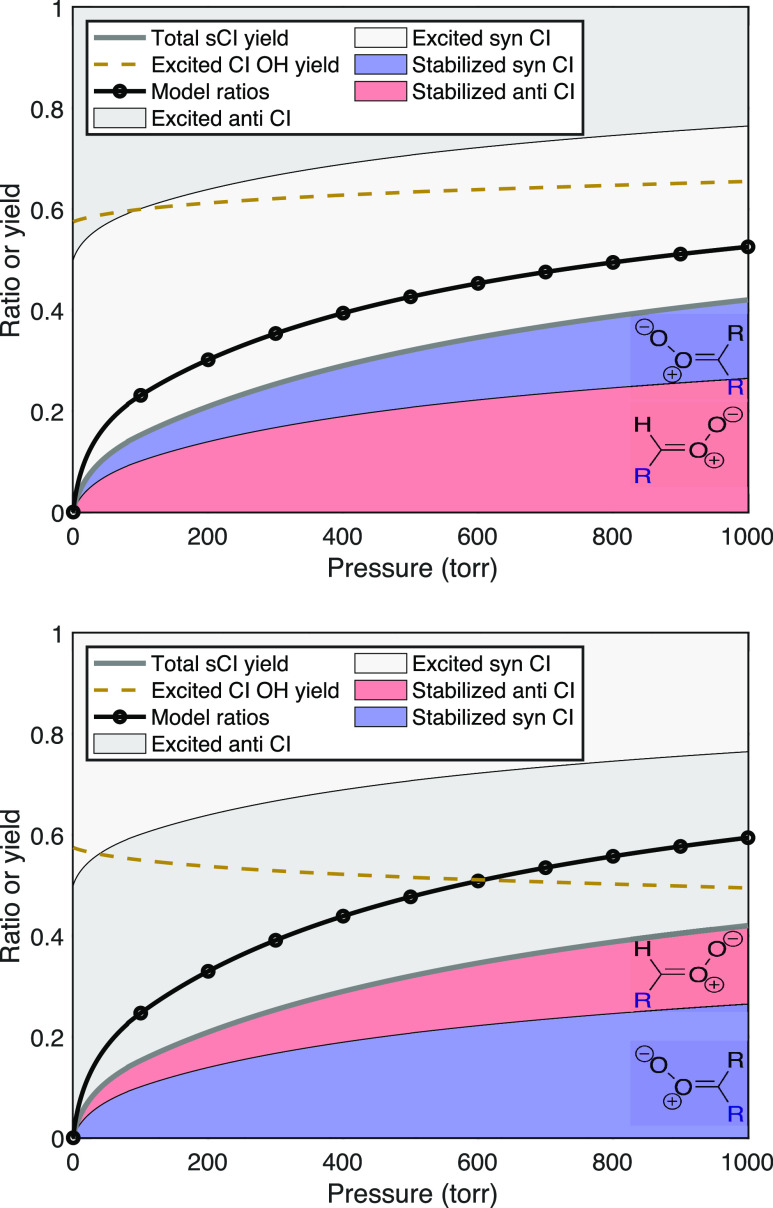
Stabilized Criegee intermediate yields, sCI, and H_2_SO_4_ signal ratios, R = *S*_scav_/*S*_tot_, for two cases with a 50:50 split between syn- and anti-intermediates. In the top case, the *anti*-conformer stabilizes first, and so the OH yield from the residual (mostly syn) excited species rises with pressure, keeping the signal ratio closer to the actual stabilization yield. In the bottom case, the *syn*-conformer stabilizes first, and so the OH yield from the residual (mostly anti) excited species decreases with increasing pressure, causing the signal ratio to rise progressively above the actual stabilization yield.

For many alkenes, ozonolysis results in a mixture of syn- and anti-Criegee intermediate conformers. For linear trans alkenes larger than *trans*–2-butene, there is little preference in the cycloreversion for the two conformers, and so a 50:50 mixture is expected and observed, whereas for *cis* alkenes, the *anti*-conformer is favored and the syn/anti ratio is roughly 20:80.^13^ With a mixture of excited Criegee intermediates that also have very different OH yields, collisional stabilization will preferentially deplete the longer lived (excited) conformer and consequently change the OH yields compared to those of the excited intermediates. This in turn will change the observed H_2_SO_4_ signal ratio. We shall refer to this longer lived excited conformer being “stabilized first”, meaning that proportionally more of the longer lived intermediates are stabilized than the shorter lived intermediates at any given pressure. Figure 3 shows the signal ratios that would be observed for a 50:50 split depending on whether the anti- (top) or syn- (bottom) intermediate is stabilized first. The overall stabilization yield remains the same as for the other examples, but the stabilization fraction is also pressure-dependent with *p*_c_^ratio^ = 500 Torr. The figure also shows the OH yield from the excited intermediates, *y*_all_^OH^, as a dashed gold curve; this rises with pressure when the anti-intermediate is preferentially stabilized and falls with pressure when the syn-intermediate is stabilized first.

Overall, these examples show that the signal ratios are strongly dependent on the number of intermediate conformers as well as their relative stabilization. For a single reaction, this means that the H_2_SO_4_ signal ratio itself does not uniquely constrain the intermediate stabilization. For the same overall stabilization, the ratios vary from 0.4 to 0.8, with feasible values over the full range. However, for a sequence of reactions, this sensitivity provides additional information. There are other a priori constraints on the prompt syn:anti ratio and the overall OH yields at different pressures, and we also expect physically sensible evolution of the physically based parameters along the sequence. These constraints will inform whether the anti- or syn-intermediates are stabilized at lower pressure (first).

## General Potential Energy Surface

**Figure 4:**
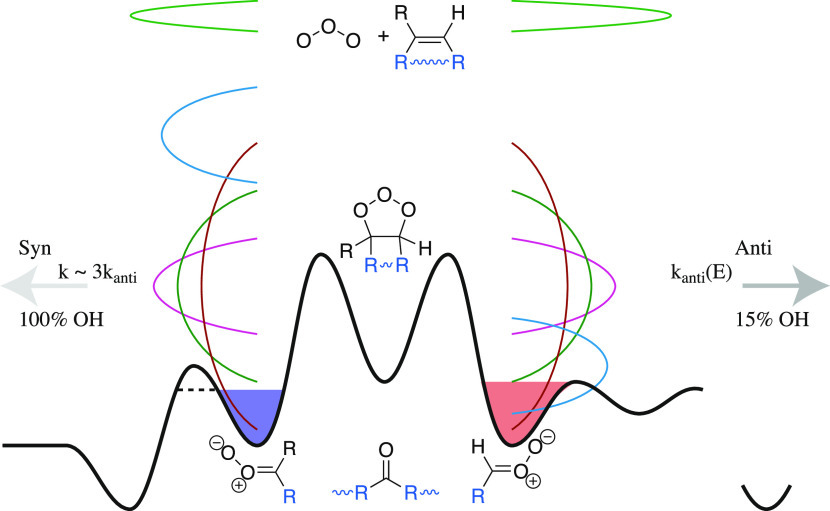
Generalized potential energy surface and energy distributions for the ozonolysis of alkenes. Ozone reacts with an alkene, shown in the center top; blue groups may be or H or R and may or may not be tethered to form an endocyclic species, shown with a wavy blue bond. This releases order 30,000 cm^–1^ (360 kJ mol^–1^) of energy, forming a 1,2,3–trioxolane primary ozonide. Two major pathways then form either syn-Criegee intermediates (to the left) or anti-Criegee intermediates (to the right). A carbonyl coproduct may or may not be attached, depending on the endocyclic tether. Both the absolute magnitude and the width of the prompt energy distribution of the Criegee intermediate products (balanced by the carbonyl coproduct and any external modes) depends on the initial alkene; distributions have colors corresponding to various alkenes throughout the paper. Some of the intermediates are stabilized, forming stabilized Criegee intermediates (sCIs), indicated with the blue and salmon filled wells. Others dissociate to form OH radicals, with either 100% yields (for syn-intermediates) or 15% yields (for anti-intermediates). Dissociation of the syn Criegee intermediates includes H atom tunneling, effectively lowering the barrier and increasing the microcanonical rate coefficients above the corresponding anti-values.

Figure 4 shows a generalized potential energy surface based on quantum chemistry^18,37^ along with the important features of the full reaction sequence presented here. The syn Criegee intermediates are to the left and the anti-Criegee intermediates are to the right of the central primary ozonide. Reactants (alkene and ozone) are central above the PES at a common energy (roughly 30,000 cm^–1^ above the Criegee intermediates). For tethered (endocyclic) alkenes such as α-pinene, the total reaction energy is retained in a single intermediate with a narrow green energy distribution, either toward syn or anti.^8,37^ For symmetrical alkenes such as *trans*–2-butene, with the wide red distributions, less than half the reaction energy remains in the internal modes of either the syn- or anti-intermediates, balanced by the similarly sized coproduct. A (potentially large) fraction of the energy goes into external modes (translation and rotation).^2,16^ The width of the distribution depends on the number of internal degrees of freedom, with larger alkenes such as 7-tetradecene, with the narrower magenta distribution contrasting with the wide *trans*–2-butene distribution. For terminal alkenes such as β-pinene, with blue product distributions, the larger intermediate (nopinone oxide with a formaldehyde coproduct) retains much more energy than the smaller intermediate (formaldehyde oxide with *a* nopinone co–product), but the two distributions have the same width. This asymmetry has been shown to dramatically affect sCI yields.^34^

Depending on the energy distribution as well as the size (number of internal modes) of the intermediates, the Criegee intermediates may be collisionally stabilized to form sCI, with stabilized wells indicated with blue (syn–sCI) or salmon (anti–sCI) fill color. Tethered sCIs could possibly isomerize to secondary ozonides,^37,45^ but here, sufficient SO_2_ scavenges any sCIs before this occurs (if it is significant).

H atom tunneling lowers the effective barrier from the syn-Criegee intermediate to a vinyl hydroperoxide (well to the left) by roughly 2000 cm^–1^.^18^ This decomposes rapidly to form OH and an organic radical; some vinyl hydroperoxide stabilization may delay this,^8,11,16^ but on the time scale of the experiments described here, we assume that nearly 100% of the syn–CI that decomposes forms OH. Most anti–CIs decompose almost exclusively via C–O–O ring closure to form a dioxirane, which proceeds to a highly excited hot acid (deep well to the right) and then to many fragments, including approximately 15% OH. This has been confirmed via direct laser-induced fluorescence (LIF) measurement of OH and OD from selectively deuterated *trans*– and *cis*–3-hexene, which show minimal kinetic isotope effects for OH (or OD) formation from vinylic hydrogens, consistent with ring closure (and not H-abstraction) being the rate-limiting step for subsequent OH formation.^13^ Vinyl substituents, such as those following isoprene ozonolysis, enrich the chemistry of anti–sCI.^46^

## Experimental Methods

We conduct experiments in a flow reactor at 295 K over a wide pressure range (50–900 Torr). The residence time in the flow system after the ozone injection is 9 s at each pressure. We described the method in detail in prior publications^35,36^ and so here present only the necessary details. Upstream, we mix the alkene, ozone, and SO_2_. For some cycles, we add propane to scavenge OH before it can react with SO_2_. We add sufficient SO_2_ to completely titrate all sCIs and OH, which we verify experimentally. In order to maintain a constant pressure and reaction time in the ion molecule reactor (IMR), we use a series of pinholes with diameters of 0.005, 0.006, 0.008, 0.010, 0.013, and 0.020 to control sample injection from the flowtube to the IMR. We set the sheath, sample, and HNO_3_ reactant, so the residence time in the IMR is the same as for typical operation at atmospheric pressure.

We use N_2_ carrier gas from a cryogenic dewar and add 50 ppm SO_2_, 550 ppm H_2_O, and well under 100 ppb alkene, along with less than 500 ppb ozone. The concentrations of the alkene, ozone, and H_2_O are low enough so that SO_2_ (and propane) are the only significant sinks of OH and sCI.^5^

At each pressure (with a given pinhole), we conduct a series of measurements with four stages. These are background (bkg); scavenger (propane) only (scav); sCI; and sCI + OH, (tot). The background stage includes N_2_, O_3_, H_2_O, and SO_2_. For the scavenger-only stage, we add 500 ppm propane to this mix—this forms a separate background for the scavenger signal because the propane can contain minute amounts of propene and thus lead to a small increase in the H_2_SO_4_ signal.^35^ The sCI stage adds alkene (with the propane), and we subtract the scavenger-only H_2_SO_4_ signal from the signal in this stage to find the background-corrected H_2_SO_4_ with the scavenger, which is the sCI signal. For the sCI + OH stage, we turn off the OH scavenger and then subtract the background stage signal to give the sCI + OH (total) signal. The signal ratio is then
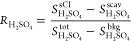
5

## Experimental Results

**Figure 5:**
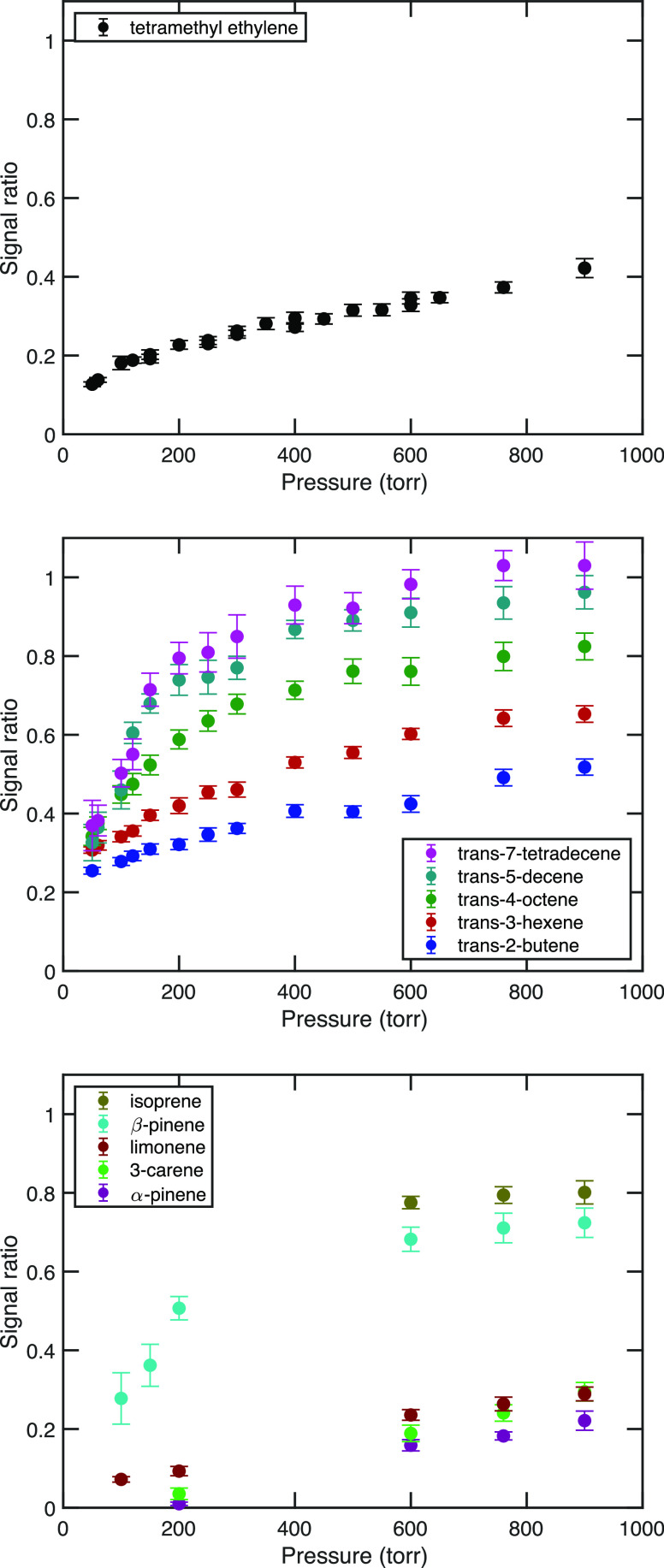
Observed signal ratios vs pressure for three classes of alkenes: (top) tetramethylethylene (TME);^35^ (middle) a series of symmetrical trans alkenes;^36^ and (bottom) a series of monoterpenes as well as isoprene. Signal ratios are related to stabilized Criegee intermediate yields but require correction for non-unit OH yields, as discussed in the text.

Figure 5 shows the signal ratios vs pressure, for three sets of alkenes: tetramethylethylene alone,^35^ symmetrical trans alkenes from *trans*-2-butene through *trans*-7-tetradecene (skipping *trans*-6-dodecene),^36^ and new observations for a set of terpenes (3-carene, α-pinene, β-pinene, limonene, and isoprene). We previously published the first two sets, and the terpene data are new.

The tetramethylethylene case is the simplest, as ozonolysis produces exclusively (syn) acetone oxide. As discussed above, this means that *y*^OH^ = 1 and the signal ratio vs pressure can be interpreted directly as the stabilization of the intermediate.^35^ The symmetrical trans alkenes form intermediates with a syn/anti ratio near 50:50,^13,36^ and the potential energy surfaces for the reaction are likely very similar for the sequence. Consequently, the major variable is the size (carbon number) of the alkene and the resulting intermediate. The number of loose vibrational modes increases by 3 with each added carbon, and so the unimolecular rate coefficients along the reaction coordinate drop dramatically.^37,47^ As a consequence, collisional stabilization at a given pressure is more efficient for larger molecules, for the Criegee intermediate but also possibly for the primary ozonide as well.^37^

The terpenes are common in the remote atmosphere as they have copious emissions from both coniferous trees (for the monoterpenes) and from deciduous trees (for isoprene).^48^ Many alkenes are also found in urban environments, where ozonolysis can be an important OH source, especially when light is low (including at night).^6^ Several structural features are relevant. All the monoterpenes here are cyclic. Three are endocyclic, containing a methyl-substituted double bond within a 6-member ring (3-carene, α-pinene, and limonene). This dramatically alters the energy distribution following cycloreversion of the primary ozonide, as rather than the two products formed from linear alkenes, only a single, tethered intermediate emerges.^37^ Consequently, all the energy from the cycloreversion exothermicity is retained by the unimolecular intermediate product, as shown by the green distributions in Figure 4. In contrast, β-pinene, the vinyl substituent in the doubly unsaturated limonene, and isoprene are exocyclic or linear alkenes and so more closely resemble the trans alkenes. Limonene also has an exocyclic vinyl group, but like all terminal C=C double bonds, its specific rate coefficient with ozone is at least a factor of 10 slower than the endocyclic double bond.^49,50^ However, these are asymmetric terminal alkenes, and so the cycloreversion forms two very different intermediates, either formaldehyde oxide, CH_2_OO, or a C_9_ (or C_4_ for isoprene) Criegee intermediate. This in turn affects the energy distribution in the intermediates, as shown by the blue distributions in Figure 4.^34^

The results in Figure 5 have various obvious features. First, all the ratios fall within the expected range, 0 ≤ *R* ≤ 1, and they span this full range. Second, the evolution makes general sense. For the homologous trans alkene sequence, the observed signal ratios increase with the carbon number at any given pressure, consistent with more efficient stabilization for intermediates with more internal degrees of freedom, as expected. The stabilization for both *trans*–5-decene and *trans*–7-tetradecene appears to be nearly complete by 1000 Torr. Third, the terpene results fall in two distinct groups, defined by the presence or absence of endocyclic double bonds. The endocyclic monoterpenes 3-carene, α-pinene, and limonene all show modest stabilization with signal ratios between 0.2 and 0.3 at 1000 Torr, and for purely endocyclic 3-carene and α-pinene, the signal ratio drops to zero near 200 Torr—with the OH scavenger present, no extra H_2_SO_4_ was formed when the terpene was added to the system at lower pressure, even though the OH yields are high for these terpenes and even though for many other alkenes shown in the figure we did observe a significant signal at lower pressure. We did observe the H_2_SO_4_ signal at low pressure from the exocyclic terpenes β-pinene and limonene; however, the β-pinene has a much larger signal ratio (lower contrast with the OH scavenger) than the other terpenes at all pressures. The limonene resembles the other endocyclic terpenes above 200 Torr but also shows a finite signal at lower pressure as well. Finally, we observed high signal ratios from isoprene over a more limited pressure range from 600 to 900 Torr.

## Interpretation

Our earlier paper describing pressure stabilization (sCI yields) in the trans alkene homologous sequence used a statistical sampling approach to separate the observed H_2_SO_4_ signal ratios (Figure 5 middle) between contributions from the syn- and *anti*-conformers.^36^

Rather than formally fitting the ratios for individual alkenes using least-squares or other optimization, here, we instead seek a consistent and physically meaningful set of parameters for the entire sequence of reactions. There are several reasons for this. First, with multiple Criegee intermediates in most cases, the parameter set is degenerate without other constraints to separate the parameters. We addressed this when first reporting the trans alkene results via a statistical search.^36^ Second, systematic errors in the data may bias any fitting because the different parameters can yield similar results (i.e., *f*_c_ and *p*_c_ parameters have high negative covariance for even one falloff curve). Finally, as we seek a set of parameters that are consistent across the entire sequence, it is not obvious how to weigh data from different reactions in the overall optimization. Consequently, we report parameters in Table 1 that give physically sensible, consistent results for the entire sequence.

**Table 1:** Model Parameters for the Pressure Dependence of Criegee Intermediate Stabilization from a Sequence of Alkenes, Based on the Measured H_2_SO_4_ Ratios in a Flow Containing the Alkene, O_3_, and SO_2_, without and with an OH Scavenger (Propane)^a^

alkene	*f*_c_	syn/anti	α_OH_^syn^	*p*_c_^syn^ (torr)	α_sCI,°_^syn^	*p*_°_^syn^ (torr)	α_OH_^anti^	*p*_c_^anti^ (torr)	α_sCI,°_^anti^	*p*_°_^anti^ (torr)
tetramethylethylene	0.3	100:0	1.0	100	0.05	0				
*trans*-2-butene	0.25	60:40	1.0	200	0.1	0	0.15	67	0.15	
*trans*-3-hexene	0.3	50:50	1.0	100	0.05	0	0.15	33	0.1	
*trans*-4-octene	0.4	50:50	1.0	30	0	40	0.15	10	0	20
*trans*-5-decene	0.5	50:50	1.0	6	0	100	0.15	2	0	40
*trans*-7-tetradecene	0.65	50:50	1.0	2.1	0	125	0.15	0.7	0	45
α-pinene	0.7	80:20	1.0	2200	0	180	0.15	733	0	180
3-carene	0.7	80:20	1.0	2000	0	170	0.15	667	0	150
limonene (endo)	0.7	0.9 × (80:20)	1.0	1800	0	170	0.15	600	0	150
limonene (exo)	0.5	0.1 × (60:40)*	1.0	40	0	75	0.15	1000	0.2	0
β-pinene	0.5	60:40*	1.0	40	0	75	0.15	1000	0.2	0
isoprene (CH_3_OO)	0.275	0.58					0.15	1000	0.3	0
isoprene (vinyl)	0.275	0.03					0	20	0	0
isoprene	0.275	0.39 × (26:74)	1.0	60	0	0	0.15	20	0	0

a Parameters are for modified Lindemann–Hinshelwood pressure falloff curves with either a zero-pressure intercept (*y*_°_) or an induction pressure *p*_°_. Parameters are given for both syn- and *anti*-conformers, produced in the indicated (syn/anti) ratio (* for the exocyclic terpenes; the indicated ratio is instead R_2_COO:CH_2_OO). Branching for isoprene based on Nguyen et al.^46^

Our main consideration is that the model parameters should vary in a physically sensible way across these reaction sequences. We also add some a priori constraints to the model. First, we assume that the prompt energy distribution of the nascent excited intermediates grows progressively narrower as the carbon number increases, as shown in Figure 4. This is because the energy is distributed statistically among the internal modes of the molecule, and statistical distributions narrow with increasing size. Most notably, the endocyclic alkenes will have a very narrow initial energy distribution in the vibrationally excited product, as all of the reaction energy will remain in the single, tethered intermediate.^8,37^ Because broadening in pressure falloff curves is related to a distribution in excited-state lifetimes (unimolecular rate coefficients), we assume that the broadening term, *f*_*c*_, will be greater for smaller intermediates with relatively wide initial energy distributions. Specifically, for linear and exocyclic alkenes, we assume that *f*_*c*_ scales with the POZ carbon number.

Second, we assume for a homologous sequence (like the trans alkenes) the characteristic pressure (the center of the falloff curve) will decrease with increasing carbon number. A consequence of very wide initial energy distributions in the intermediates will be some intermediates being “born cold” with energies below the critical energy for decomposition, leading to a nonzero intercept, *y*_°_. In contrast, intermediates with very narrow initial energy distributions and especially also very high initial internal energy will display an “induction pressure”, *p*_°_, with essentially no stabilization below that value (as with the endocyclic monoterpenes). Earlier observations of sCI formation using FTIR measurement of secondary ozonides formed using hexafluoroacetone as a scavenger also showed no sCI yields for cyclohexene ozonolysis up to 1 atm.^51^ Finally, based on considerations described below as well as earlier work, we assume that the *anti*-conformers stabilize earlier (*p*_c_^anti^ < *p*_c_^syn^).

The end result of this analysis is the parameter set presented in Table 1 and shown phenomenologically in Figure 4. We are able to reasonably reproduce the experimental signal ratios versus pressure with very few mathematical degrees of freedom in the modified Lindemann–Hinshelwood function. Most notably, the characteristic pressure for anti–CI stabilization is consistently one-third the value for syn–CI for any given alkene; this is consistent with the microcanonical rate constants for the reaction over the syn–CI barrier being 3 times higher than the corresponding rate constants (at any given energy) for the anti–CI, as shown in Figure 4, so 3 times the pressure (and collision frequency) causes equivalent stabilization. The other parameters also evolve smoothly for the alkene sequences. We start by assuming a steady evolution in *f*_c_, for the linear and exocyclic alkenes, increasing (toward 1.0) as the POZ carbon number and thus the nascent energy distribution narrows. This is also significantly closer to 1.0 for the endocyclic terpenes, where the nascent distribution is sharp. The intercepts (α_°_ or *p*_°_) also evolve smoothly as the energy distribution narrows, moving systematically from relatively large zero-pressure yields to relatively large induction pressures.

Below, we discuss the results for groups of alkenes.

### Hexenes

**Figure 6:**
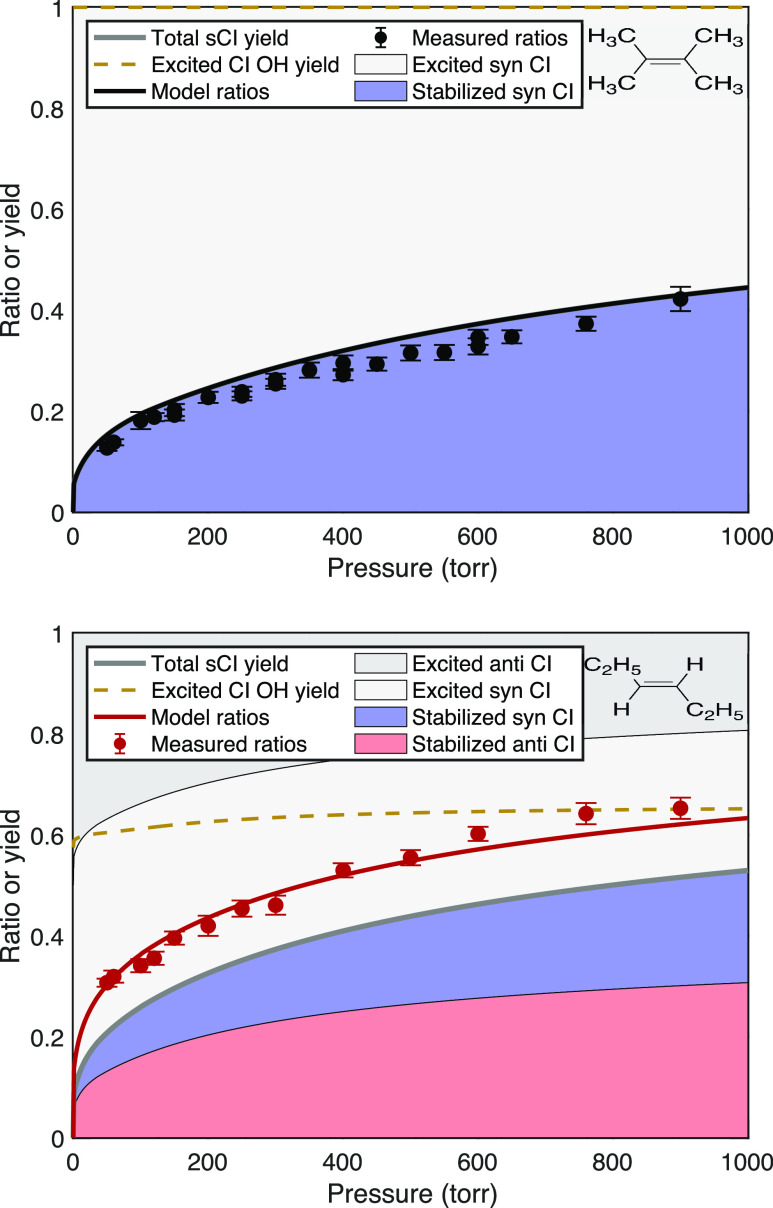
Pressure stabilization for tetramethylethylene (top) and *trans*-3-hexene (bottom) showing the observed and model signal ratios as well as the fractions of syn- and anti-Criegee intermediate conformers that are either stabilized (in salmon and blue) or excited (gray). The dashed gold curve shows the OH yield from the excited intermediates.

We anchor our analysis with the two hexenes tetramethylethylene and *trans*–2-hexene, with results shown in Figure 6. In addition to being symmetrical, the tetramethylethylene system is well constrained. The unimolecular rate coefficients for OH yields from acetone oxide have been measured via selective infrared laser excitation and subsequently OH actinometry,^17^ and the unimolecular rate coefficients for acetone oxide decomposition have been calculated at a high level of theory, including semiclassical transition-state theory tunneling corrections.^18^ The measured and calculated unimolecular rate coefficients match well, and associated master equation simulations of acetone oxide stabilization following tetramethylethylene ozonolysis using those rate coefficients also match the observed pressure-dependent stabilization shown in Figure 6. The model parameters in Table 1 shown in Figure 6 are indistinguishable from the master equation results using experimentally and theoretically verified unimolecular rate coefficients.^18^

Several findings from the master equation simulation are germane for our analysis beyond the excellent agreement between those results and our modified Lindemann–Hinshelwood function. First, H atom tunneling is essential to the agreement, both for our results in Figure 6 and also for the agreement between experimental and RRKM unimolecular rate coefficients.^18^ Master equation simulations with and without tunneling show a reaction flux with a peak energy that is approximately 2000 cm^–1^ lower when tunneling is considered.^18^ This is functionally equivalent to lowering the effective transition-state energy by 2000 cm^–1^. Second, the initial energy distribution in the excited acetone oxide in the master equation simulation was quite broad, spanning nearly 4000 cm^–1^ full width at half maximum, consistent with Figure 4.

Other quantum chemical and master equation calculations for substituted cyclohexenes found that the transition state for H atom transfer from the *syn*-conformer is approximately 2000 cm^–1^ lower than the transition state for the *anti*-conformer to undergo a ring closing reaction to form a substituted dioxirane;^37^ however, these calculations did not consider tunneling. Consequently, the effective transition-state energies for loss of the *syn*- and *anti*-conformers are similar after considering tunneling for the H atom transfer.

As detailed in our prior discussion of the trans alkene sequence, we can fit H_2_SO_4_ signal ratios with a model assuming either that the *syn*- or *anti*-conformer stabilizes first (with a lower *p*_c_). However, it is not possible to find a sensible fit for the ensemble of reactions when assuming that the syn conformers stabilize first.^36^ As shown in Figure 6, the ratio data for *trans*–3-hexene are fit reasonably, assuming 50:50 syn:anti ratio with the *anti*-propanal oxide conformers (salmon) stabilizing first and the *syn*-conformers (blue) stabilizing second, with parameters that closely match the acetone oxide isomer. Because the anti–CI stabilizes first, the OH yields from the excited intermediates rise progressively with pressure. Overall, the model parameters include a nonzero fraction of sCI at zero pressure, roughly 5% of the syn and 10% of the *anti*-conformers.

### Trans Alkenes

**Figure 7:**
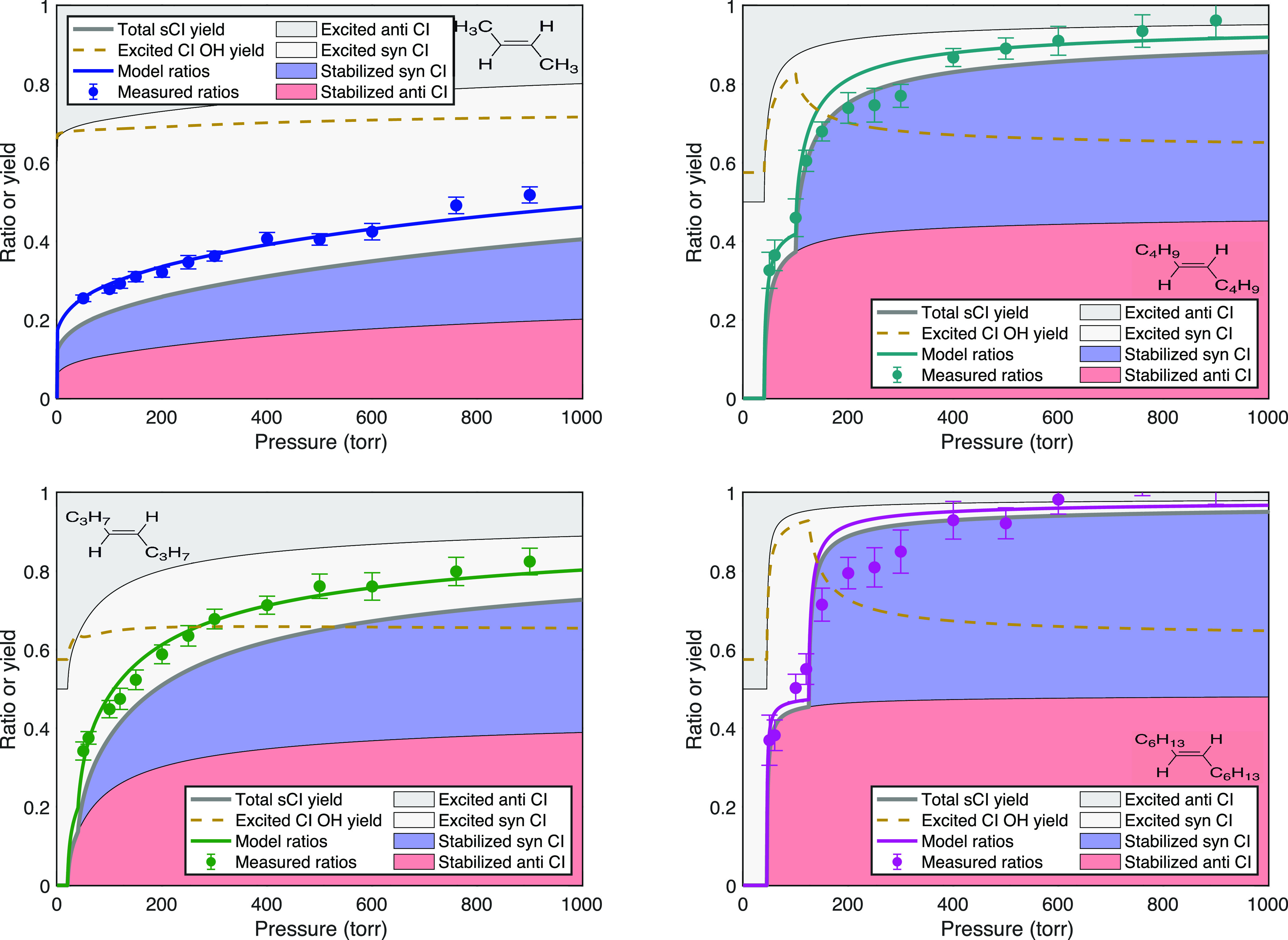
Pressure stabilization for trans alkenes showing the observed and model signal ratios as well as the fractions of syn- and anti-Criegee intermediate conformers that are either stabilized (in salmon and blue) or excited (gray). The dashed gold curve shows the OH yield from the excited intermediates. Stabilization grows progressively with the carbon number, with larger alkenes also showing a significant induction pressure, followed by sharply rising yields at higher pressure.

The model fits for the remaining trans alkenes are shown in Figure 7, with *f*_c_, *p*_c_, and α_°_ or *p*_°_ evolving systematically. The trans–2-butene falloff is broad and shows a significant zero-pressure intercept for both conformers, consistent with our assumptions. All the larger trans alkenes show signs of an induction pressure, with stabilization rising rapidly for pressures above that value; however, we did not conduct measurements below 50 Torr due to experimental limitations (and the pressure dependence of the SO_2_ + OH reaction itself), and so this induction pressure is inferred rather than observed (unlike the α-pinene and 3-carene, where we observe the yields dropping to zero below 200 Torr).

Given the consideration that the parameters in Table 1 evolve sensibly across the sequence, the results are satisfactory even for *trans*–5-decene and *trans*–7-tetradecene. Our treatment of the induction pressure is surely an oversimplification. However, the observed ratios do rise very sharply with pressure above the apparent threshold, and they rise to nearly 1.0 for *trans*–7-tetracecene. The model overestimates the measured ratios between 150 and 350 Torr (this appears for *trans*–4-octene to a lesser extent) but otherwise fits the data well. Especially for *trans*–7-tetradecene, there may be nonzero stabilization of the primary ozonide before cycloreversion,^37^ which would further complicate the overall pressure dependence for these larger alkenes.

### Terpenes

The terpene data are new experimental results. The measured H_2_SO_4_ signal ratios depend strongly on the terpene structure, and so we shall discuss them in sequence.

#### Endocyclic Monoterpenes

**Figure 8:**
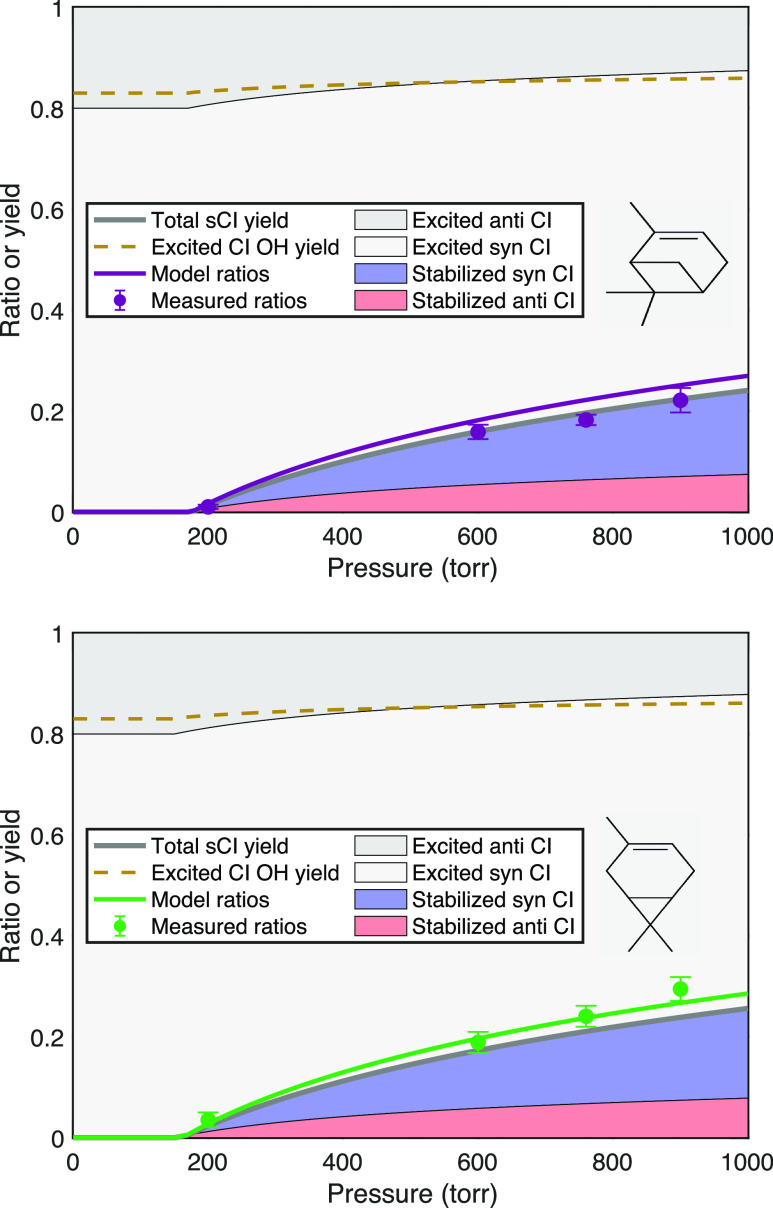
Pressure stabilization for endocyclic monoterpenes (α-pinene and 3-carene) showing the observed and model signal ratios as well as the fractions of syn- and anti-Criegee intermediate conformers that are either stabilized (in salmon and blue) or excited (gray). The dashed gold curve shows the OH yield from the excited intermediates. Both terpenes show very similar stabilization, with no sCI yield below an induction pressure just below 200 Torr and a slowly rising stabilization reaching roughly 15% at 760 Torr.

The endocyclic monoterpenes (α-pinene and 3-carene) shown in Figure 8 have very similar signal ratios with slightly higher values for 3-carene. Both are known to have a roughly 80:20 syn/anti ratio (including 3 distinct syn isomers) with overall OH yields near 0.8.^5,52^ Even with the *anti*-conformer stabilizing first, the preponderance of syn conformers suggests a substantial stabilization of those as well, with the exact split remaining uncertain. The overall recommended sCI yield is 0.18 ± 0.05 for α-pinene at 298 K and 760 Torr,^5^ which includes consistent results based on hexafluoroacetone scavenging,^51^ absolute H_2_SO_4_ formation,^53^ and differential SO_2_ and O_3_ consumption during ozonolysis.^54^ This last study also varied H_2_O to estimate that roughly 40% of the sCIs at 760 Torr have relatively high reactivity with water (compared to SO_2_) and so are consistent with anti–CI.^54^

Compared to the linear alkenes, the characteristic pressure for anti–CI stabilization shown in Table 1, *p*_c_ ≃ 600 Torr, is much greater. The reason is straightforward. The single, tethered product retains all the excitation energy, including that released into external modes during fragmentation. This leaves more than twice as much total energy in the excited intermediate (with a very narrow energy distribution). Although RRK theory is not accurate, it provides the qualitative explanation for the consequences of this added energy.^55,56^ The RRK unimolecular rate coefficients are given by the fractional excess energy raised to a high power related to the effective number of internal modes (very roughly 3*N* – 6, where *N* is the number of heavy atoms).
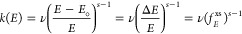
6By more than doubling the fractional excess energy compared to an equivalent Criegee intermediate from a linear alkene (which would be eicosene), with an effective number of modes between 10 and 20, the characteristic pressure for the C_10_ intermediate will be orders of magnitude higher than even that for the C_7_ intermediate from the linear alkene. The substantial induction pressure near 150 Torr for both α-pinene and δ-3-carene is also consistent with the high degree of nascent chemical activation.^8,37^ Finally, the parameters in Table 1 shown in Figure 8 give a 30:70 split for anti–sCI vs syn–sCI at 760 Torr, which is reasonably consistent with the literature estimates based on the competition between SO_2_ and H_2_O.^54^

#### Exocyclic Monoterpenes

**Figure 9:**
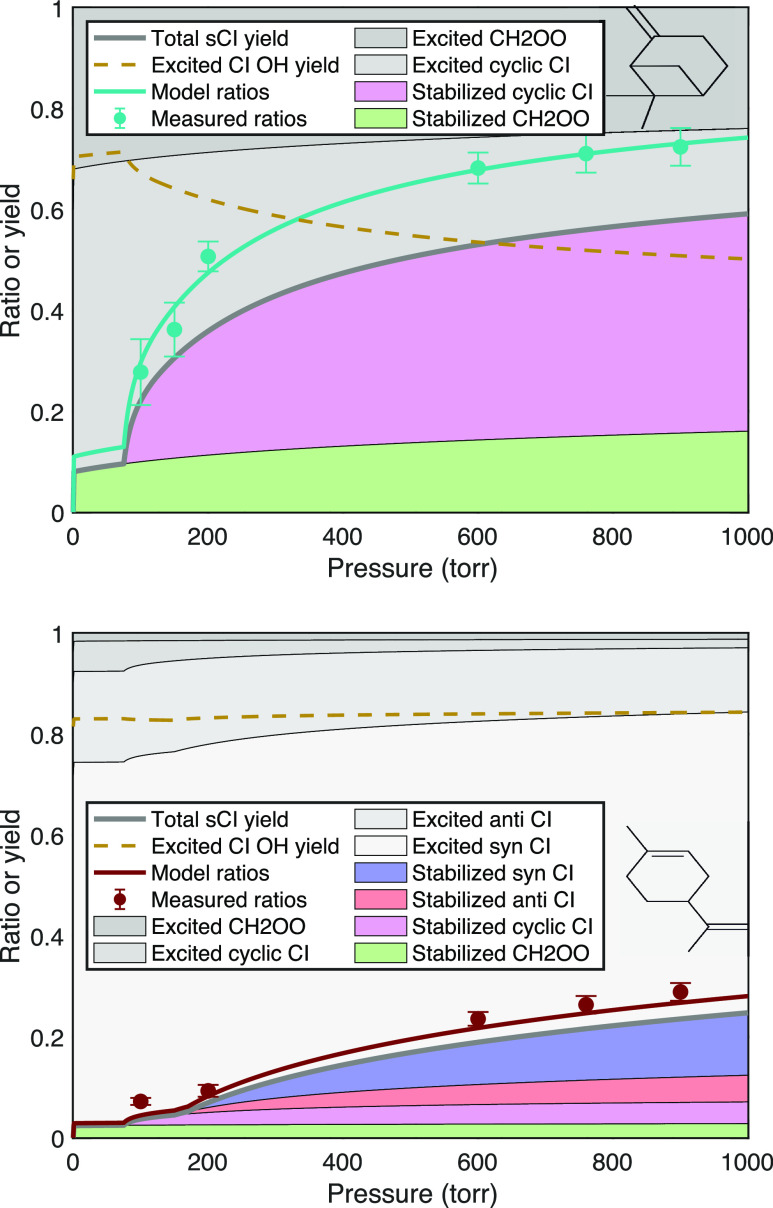
Pressure stabilization for monoterpenes β-pinene and limonene, showing the observed and model signal ratios. The dashed gold curve shows the OH yield from the excited intermediates. Exocyclic β-pinene is an asymmetrical terminal alkene, with most stabilization occurring in nopinone oxide (cyclic, magenta). Limonene, with two double bonds, is fit well as a reactivity-weighted combination of α-pinene and β-pinene.

The results for limonene and β-pinene are shown in Figure 9. The β-pinene signal ratios versus pressure are substantially larger than for the endocyclic monoterpenes. There are two reasons for this. First, the exocyclic double bond cleaves into two separate products, as with the linear alkenes. This reduces (and widens) the internal energy in the two products. However, both limonene and β-pinene have terminal double bonds, so instead of forming products with identical carbon numbers, these reactions form a C_1_ product (CH_2_O or CH_2_OO) and a C_9_ product (a ketone or ketone oxide). As shown in Figure 4, we expect the C_9_ products to have much more internal energy than the C_1_ products, with a wider energy distribution than the very narrow distribution in the single product from the endocyclic terpenes.

The importance of the asymmetry to the average energy in the nascent intermediates has been discussed for terminal alkenes,^34^ but here, we suggest that the width of that distribution is also important. The width of the initial energy distribution should resemble that of *trans*–5-decene, also C_10_, and so we assume the same *f*_c_ value. The CH_2_OO will have relatively low energy, but it is also extremely difficult to stabilize with only three heavy atoms; therefore, we anticipate a relatively large portion to be “born cold” but only a modest increase above this zero pressure initial sCI yield for CH_2_OO. The sharp increase in our observed signal ratio with pressure thus instead strongly indicates increasing nopinone oxide stabilization.

Overall, we find a modest low-pressure sCI yield with progressive stabilization of nopinone oxide above some induction pressure. This will cause the OH yield to drop with increasing pressure, consistent with the lower observed OH yields from β-pinene.^5^ At 760 Torr, our parameters suggest a yield of 0.15 for anti–sCI (CH_2_OO) and 0.40 for syn–sCI (nopinone oxide) for an overall sCI yield of 0.56. This is consistent with the recommended value of 0.55 ± 0.10,^5^ and our 27:73 branching is broadly consistent with the literature as well. The competition between SO_2_ and H_2_O suggests a 40:60 split between anti–sCI and syn–sCI;^54^ FTIR measurements give yields of 0.05 for CH_2_OO and 0.36 for nopinone oxide.^57^ Other estimates based on changes in nopinone yields with varying H_2_O also find similar branching.^54^

The limonene data resemble the other endocyclic terpenes but with the complication that limonene also has an exocyclic vinyl group. The ozonolysis rate coefficient for this double bond is thought to be 30 times slower than the rate coefficient for the endocyclic double bond,^5,49^ but some products from this secondary pathway are expected. We thus expect the overall yields from limonene to be close to a reactivity-weighted sum of the yields from 3-carene and β-pinene. A model with 30:1 ratio of ozonolysis rate coefficients using the parameters from α-pinene and β-pinene does not reproduce the finite yields observed below 180 Torr; even the model shown in Figure 9 with 10:1 rate coefficient ratio falls slightly below the 100 Torr measurements, but a faster rate coefficient for the terminal double bond is implausible.

Because of the two double bonds, comparison to the literature requires caution. Our data are driven by production rates with little reagent depletion and therefore are weighted heavily toward the more reactive (endocyclic) double bond in limonene. Our measured ratios are broadly consistent with the expected (weighted) sum of the two pathways suggested by α-pinene and 3-carene on the one hand and β-pinene on the other hand.

Comparison with other studies requires careful consideration of the overall extent of the reaction in those studies. The study of Newland et al.^54^ found an overall sCI yield of 0.27 based on SO_2_ loss; while it could be argued that traces of Δ*S*O_2_/ΔO_3_ at different RH show signs of curvature compared to more linear traces for α-pinene and β-pinene (and thus, lower sCI yields at a lower extent of reaction), this is far from conclusive. The study of Sipila et al. also was carried out in a flowtube with limited reagent depletion,^53^ and while our overall sCI yields of 0.20 at 760 Torr are broadly consistent with their reported yields of 0.27 ± 0.12, we see very similar signals for limonene compared to α-pinene and 3-carene, whereas they report yields almost a factor of 2 higher. While our absolute sCI yield estimates require independent constraints on the average OH yields from excited CI, our precision is very high; the results in Figure 5 strongly suggest that the sCI yields vs pressure for α-pinene, 3-carene, and limonene are very similar, with only the lowest pressure contribution from CH_2_OO distinguishing limonene.

#### Isoprene

**Figure 10:**
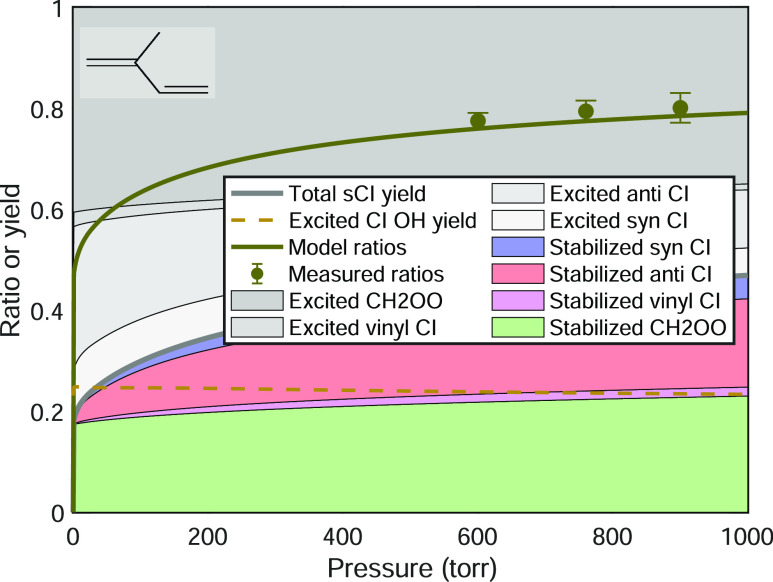
Pressure stabilization for isoprene, showing the observed and model signal ratios. Branching among the various Criegee intermediates is from Nguyen et al.^46^ The dashed gold curve shows the OH yield from the excited intermediates.

Finally, we measured stabilization following isoprene ozonolysis at 600, 760, and 900 Torr, as shown in Figure 10. The signal ratios are large, and again, we expect a portion of the CH_2_OO to be formed stable but with modest subsequent pressure dependence. We thus expect most of any pressure dependence to arise from stabilization of methacrolein oxide and methyl vinyl ketone oxide. However, both these C_4_CI have *syn*- and *anti*-conformers, and in each case, one of the syn conformers faces a vinyl group. The result is a rich mechanism that has been the subject of extensive research (although with most experiments at 1 atm pressure).^5,46^

The contribution of our work is three signal ratios versus pressure, whose interpretation is contingent on both branching and OH yields. Therefore, we shall assess consistency rather than (over) interpreting these new data, using parameters from the comprehensive mechanism of Nguyen et al.^46^ First, we assume a CH_2_OO/C_4_CI branching of 58:42, Next, we divide the C_4_CI into three groups: 24% syn–CI (making vinyl hydroperoxide and 100% OH); 69% anti–CI (all conformers that make dioxirane and 15% OH); and 7% vinyl–CI (making dioxol and no OH). We assume that 50 ppm SO_2_ is sufficient to react even with these CI values when and if they are stabilized.

Adding to literature constraints, we draw on the expected systematic evolution of parameters in Table 1. First, the overall width of the nascent energy distributions determines *f*_c_, based on the size of the POZ. We thus assume that *f*_c_ = 0.275. Second, the asymmetry will result in a substantial fraction of CH_2_OO being born cold. This is a balance between a broader nascent distribution and less profound asymmetry than β-pinene, so α_sCI,°_^CH_2_OO^ = 0.3 with the same *p*_c_^CH_2_OO^ = 1000 Torr. The relatively energy-rich C_4_CI has *p*_c_^syn^ = 60 Torr and *p*_c_^anti^ = *p*_c_^vinyl^ = 20 Torr. With carbon numbers near where the *x*- or *y*-intercepts vanish, we assume that these are all zero. The overall model function in Figure 10 agrees well with our observations, which we conclude are consistent with the Nguyen et al. mechanism.^46^ In addition to the observed ratios, the model also has a low-pressure OH yield of 0.25 with a modest pressure dependence, which is consistent with accurate low-pressure OH yields based on the calibrated laser-induced fluorescence.^58^

## Implications for Reaction Dynamics

The full ensemble with self-consistent stabilization parameters provides a test bed for the important features of chemical reaction dynamics that are in play in this highly exoergic reaction. These in turn represent constraints for dynamic calculations, such as master equation simulations. Specifically, we find that the narrowing of the statistical energy distribution in bimolecular products with increasing carbon number is an important feature necessary to support the fully consistent set of parameters and that a consequence of this is the transition from a fraction of the nascent intermediates being formed below the critical energy for the subsequent reaction (“born cold”) in the smaller systems to subsequently developing a threshold pressure for any significant stabilization (“induction pressure”) in the larger systems. The most dramatic example is the endocyclic alkenes and their induction pressures near 200 Torr.

The phase space would be generalizable with a few more reaction sequences. Symmetrical *cis* alkenes would better constrain the syn:anti ratios. A systematic sequence of terminal alkenes would constrain the asymmetric energy distributions. Sequences of substituted cyclohexenes (4,5–dialkyl–cyclohexenes with and without methyl groups at the 1 and 2 positions as well) would further constrain the induction pressures in the endocyclic systems.

## Atmospheric Implications

Collisional stabilization of Criegee intermediates following ozonolysis of alkenes is required for subsequent bimolecular reactions of stabilized Criegee intermediates. It is therefore important to understand and generalize this stabilization across the full spectrum of alkenes that are important in the atmosphere (from natural terpenoids to urban olefins). Furthermore, the conformation of these stabilized Criegee intermediates is important. The anti-sCIs are more reactive with water vapor and less reactive with SO_2_ compared with the syn-sCI.^20^ Therefore, the potential importance of the sCI as a selective oxidant for SO_2_ (enhancing gas-phase H_2_SO_4_ production compared to OH formation) depends on this. Our findings here confirm that proportionately more anti-sCIs are likely to be formed at any given pressure, and we provide parameters to individually estimate the yields from both pathways. A second important aspect is the unimolecular lifetimes of the sCI, as subsequent thermal reactivation is likely in most cases to lead to the same products observed from the nascent highly vibrationally excited intermediates. In general, this unimolecular decomposition will be more competitive with the bimolecular sinks at higher temperature because it has a much higher activation energy than the bimolecular reactions. However, terpene emissions, for example, also increase with temperature, so the overall importance of sCI chemistry as a function of the season as well as altitude remains interesting.

## Conclusions

The combination of pressure stabilization experiments, measurements on ground-state Criegee intermediates, and dynamic calculations constrained by quantum chemistry has been highly fruitful in the past.^18^ The stabilization results presented here, especially with their generalized parameters, provide a richer context for future studies. Specifically, we find that symmetrical systems, with roughly equal production of *syn*- and *anti*-conformers and roughly equal nascent energy in each, show preferential stabilization of the anti-intermediates with a characteristic falloff pressure, *p*_c_, consistently a factor of 3 lower than for the syn-intermediates. The simple interpretation is that the unimolecular rate coefficients for decomposition at high vibrational energies are roughly a factor of 3 higher for the *syn*-confirmers at any given energy. We also find evidence for narrowing of the nascent energy distribution with increasing carbon number. It remains interesting at what carbon number stabilization of primary ozonides becomes significant at atmospheric pressure, and it is still unresolved whether secondary ozonide formation in the endocyclic alkenes is competitive after stabilization.^37,45,59^

The terpene and isoprene results presented here fall in line with the much simpler model systems presented earlier, suggesting that a complete set of parameters can inform sCI formation, including the two very different conformations across the range of terpenes found in the atmosphere. This may be important for regions of the atmosphere combining high biogenic emissions and relatively high SO_2_, where H_2_SO_4_ formation from sCI + SO_2_ may contribute to new particle formation.^27,60^

These measurements are based entirely on the ratios of H_2_SO_4_ formed with and without an OH scavenger. This is convenient as it avoids any requirement for absolute calibration and is also relatively insensitive to losses such as wall loss; however, future experiments would be enhanced with complementary measurements of reaction products associated with the different (syn and anti) conformers. Finally, the collisional stabilization addressed here is complementary to and benefits from the fundamental understanding gained by direct observation of reaction dynamics for ground-state Criegee intermediates that has emerged over the past decade.^20,31^

## Supporting Information Available

Ratios of H_2_SO_4_ without and with an OH scavenger vs pressure for all alkenes (XLSX)
